# Fire blight cases in Almaty Region of Kazakhstan in the proximity of wild apple distribution area

**DOI:** 10.1007/s42161-023-01416-y

**Published:** 2023-06-12

**Authors:** Elina R. Maltseva, Galiya A. Zharmukhamedova, Zhulduzay K. Jumanova, Dinara A. Naizabayeva, Zhanna A. Berdygulova, Karina A. Dmitriyeva, Botakoz Tezekbayeva, Altyn Khassein, Yuriy A. Skiba, Natalya P. Malakhova, Gulnara A. Ismagulova, Fabio Rezzonico, Theo H. M. Smits

**Affiliations:** 1 Institute of Molecular Biology and Biochemistry, Almaty, Kazakhstan; 2 Almaty Branch of National Center for Biotechnology, Almaty, Kazakhstan; 3 Tethys Scientific Society, Almaty, Kazakhstan; 4 Environmental Genomics and Systems Biology Research Group, Institute of Natural Resource Sciences, Zürich University of Applied Sciences (ZHAW), Wädenswil, Switzerland

**Keywords:** Epidemiology, *Erwinia amylovora*, Genotyping, *Malus sieversii*, Wild apple trees

## Abstract

Fire blight caused by *Erwinia amylovora* reached Kazakhstan in 2008. Here, the disease poses a threat not only to agricultural production of apples and pears, but also to the forests of wild *Malus sieversii*, the progenitor of most domesticated apple varieties worldwide. In the period 2019–2021, the spread of fire blight in the growth area of wild apples was limited by the weather conditions. In 2022, late spring and early summer were characterized by increased rainfall and moderate temperatures favorable for the disease. The goal of this study was to monitor the distribution of fire blight in private households and small orchards in the zones adjacent to wild apple distribution areas. A total of 91 samples with fire blight-compatible symptoms were collected from cultural apples (68), wild apple (10), pear (5), hawthorn (7), and quince (1) in south-eastern and eastern Kazakhstan, resulting in 21 isolates (one from pear, one from quince, and 19 from apple) of *E. amylovora*. All isolates belonged to the archetypal CRISPR genotype A. Considering the relative proximity of the infections to the forests of wild *M. sieversii*, additional measures for fire blight control and prevention will have to be implemented, including state monitoring of the wild apple forests for disease symptoms and awareness campaigns for specially protected natural territories that safeguard *M. sieversii*, as well as for local pomaceous-fruit growing communities.

## Introduction

Fire blight caused by the bacterium *Erwinia amylovora* (Burrill) is a serious disease of apples, pears, and other plants of family Rosaceae, especially of the subfamily Maloideae (Van der Zwet & Keil 1979; Vanneste 2000). Since it was first described in the USA around 1780, fire blight has expanded its distribution area to New Zealand in 1920, reaching Europe in 1957 (Thomson 2000) and continuing its expansion to the east to Central Asia (Djaimurzina et al. 2014; Gaganidze et al. 2021; Kurz et al. 2021), while simultaneously spreading from North America to South-East Asia (Myung et al. 2016), adding new countries in its distribution area in the last decades.

The introduction of fire blight in Kazakhstan, first registered in 2008 (Drenova et al. 2012), was facilitated by massive import of planting material from Europe in the frame of the state program aimed at the increase of cultural apple plantations, which subsidized purchase of imported planting stock of fruit trees for intensive orchards’ development. Along with weak quarantine control of planting material for fire blight, the massive inflow of plants imported from countries with registered fire blight disease (over 10 million seedlings and stocks of apple, pear, and quince in 2008–2015) resulted in a significant spread of the disease in the Almaty fruit zone (Djaimurzina et al. 2014; Umiraliyeva et al. 2021). This, in turn, promoted development of state programs for fire blight monitoring and prevention, including stricter import border control and allocation of funds for chemical treatment (such as phytobacteriomycin) and reimbursements for cut trees. These efforts are ongoing, and in 2021, the state-funded chemical treatment of 728.5 hectares (ha) for fire blight prevention. At the same time, the roadmaps for 2021–2025 agricultural investment projects aim at further expansion of intensive orchards, approving 32 projects for a total of 100 billion tenge (about 2,150,000 US dollars) and creating another 866 ha of apple orchards in 2021 (https://primeminister.kz/ru/news/reviews/itogi-razvitiya-sfery-selskogo-hozyaystva-za-2021-god-i-plany-na-predstoyashchiy-period-22422). The increased demand for apple seedlings to cover such a large area exceeds the capacity of local plant nurseries to deliver the required numbers of plants, and the import of plant material increases the risk of fire blight in the upcoming years.

Whereas most countries have pome fruit trees only in orchards and gardens, in Kazakhstan, wild apple forests are present. The country is home to *Malus sieversii*, a wild apple species that is the progenitor of the domesticated apple (Cornille et al. 2012; Harris et al. 2002; Velasco et al. 2010). The species is listed as “vulnerable” in the Red List of Threatened Species of the International Union for Conservation of Nature (IUCN) (https://www.iucnredlist.org/species/32363/9693009) and its study and conservation attract both domestic and international attention, given the importance of safeguarding the original gene pool (Dzhangaliev 2007, 2010).

The relative proximity of the wild apple forests to the cultural pome fruit varieties increases the risk of fire blight spread to the wild trees, especially if mediated by birds, insects, and wind (Anonymous 2022), or by human activities in the adjoining areas. The threat of fire blight spreading to the wild apple forests needs to be considered and a regular monitoring of the situation is deemed essential (Djaimurzina et al. 2014; Doolotkeldieva et al. 2021; Feurtey et al. 2020; Umiraliyeva et al. 2021). Trees of *M. sieversii* may generally be more prone to this disease compared to cultural varieties as, due to the absence of the pathogen in this region, it could not build up a natural resistance. Therefore, some studies have shown increased severity of fire blight once *E. amylovora* successfully has infected trees of this species (Harshman et al. 2017; Luby et al. 2002).

## Materials and methods

### Field observations and sampling

The objects of study were culturally grown apple, pear, quince and hawthorn trees, and wild trees of *M. sieversii*. Geographic coordinates were determined with Garmin ETREX 32x (Vancouver, WA, USA). Maps were created using BatchGeo software (Vancouver, WA, USA).

Observations of trees for fire blight symptoms were carried out on all four sides of the tree crown in two diagonals of the forest/orchard or on separate tree stands. The crowns, trunks, branches, and flowers of the trees were carefully examined for the presence of fire blight-compatible symptoms. In the case of medium- or large-scale cultural plantations, 10–20 trees were inspected. In small plantations and private households with less than 10 trees, all of the trees were inspected.

Symptoms were evaluated on a 6-point scale: 0 healthy tree; 0.1 barely visible signs of illness; 1 the initial stage of disease manifestation (single wilting and blackening of the flowers, twisting, and browning of shoots and leaves are noticeable); 2 flowers, shoots, and leaves are affected more than 10%; 3 damage to the bark of branches, trunks, fruits (bacterial exudate is released on the affected areas); 4 more than 75% of the crown is burnt, as after a fire; 5 tree dead from disease.

The percent of distribution (frequency of occurrence) of the disease was calculated by the following formula:

$$P= \frac{H\cdot 100}{N}$$

where *P* referred to the prevalence or frequency of disease, %; *H* number of infected trees; *N* number of studied trees

The degree of disease development is calculated by the following formula:

$$R\mathit=\frac{\mathit\Sigma\mathit{(a\cdot b)}\mathit\cdot\mathit{100}\mathit\;}{\mathit N\mathit K}$$

where *R* referred to the level of disease development, %; *Ʃ* the sum of multiplication of *a* and *b*; *a* the number of trees with same disease lesions; *b* lesion score corresponding to this symptom; *N* total number of trees; and *K* the highest score of the lesion scale.

### Bacteriology and immunochromatography

Samples for laboratory analyses (flowers, shoots, fruitlets, stem segments) were taken from symptomatic and asymptomatic trees and processed according to standard on diagnostics of the European and Mediterranean Plant Protection Organization (EPPO) (Anonymous 2022). Asymptomatic samples were enriched in liquid King’s B media (King et al. 1954) prior to plating and serological/molecular tests. Enriched cultures were plated on King’s B solid medium and NSA medium in accordance with the same international guidelines.

Immunochromatographic tests were conducted with the Ea AgriStrip (Bioreba, Reinach, Switzerland) (Braun-Kiewnick et al. 2011) according to manufacturer’s instructions. Nucleic acids were extracted with the EasyPure Bacteria Genomic DNA Kit (TransGen Biotech, Beijing, China) according to manufacturer’s instructions.

Immature pear fruit assays were carried out according to the method described by Doolotkeldieva et al. (Doolotkeldieva et al. 2021). In short, immature pear fruits (4–5 cm in diameter) were surface sterilized with 0.5% sodium hypochlorite and thoroughly rinsed in water, then cut in 3–4 mm slices and inoculated with 10 µl of 10^6^ cells ml^−1^ pure culture suspension. Results were observed 4–5 days after growth in a humid chamber at 25 °C, subcultured on agar and re-checked with PCR.

### Molecular test and CRISPR genotyping

Molecular tests were carried out with the protocols of Stöger et al. (2006) for conventional polymerase chain reaction (PCR) on an Eppendorf Mastercycler X50s (Eppendorf, Hamburg, Germany) and the protocol of Pirc et al. (2009) for real-time PCR on QuantStudio 5 (Applied Biosystems, Waltham, MA, USA).

For characterization of the strains, we used the approach that targets spacer 1029 in CRISPR repeat region 1 (CRR1) and allows discrimination between two ancestral populations first colonizing Europe in a single PCR assay (Kurz et al. 2021). The list of primers used for the study is provided in Table 1.

**Table 1:** List of primer sequences used for *E. amylovora* diagnostics and CRISPR genotyping

Name	Sequence	Amplicon size	Target	Reference
PEANT1	5′- TAT CCC TAA AAA CCT CAG TGC -3′	391 bp	Plasmid pEA29	(Stöger et al. 2006)
PEANT 2	5′- GCA ACC TTG TGC CCT TTA -3′			
Ams116F	5′- TCC CAC ATA CTG TGA ATC ATC CA -3′	74 bp	*ams*C gene	(Pirc et al. 2009)
Ams189R	5′- GGG TAT TTG CGC TAA TTT TAT TCG -3’			
Ams141T	5′-FAM- CCA GAA TCT GGC CCG CGT ATA CCG -TAMRA-3′			
C1f04	5′- CGA TCA ACC TGT TTT TCA GTA GGT -3′	215 or 276 bp	Spacer 1029 of CRR1	(Kurz et al. 2021)
C1r09	5′- CCG CCG AGA CAA CCG GCT ATC C -3′			

Electrophoresis of the PCR products was conducted on 1.5% agarose gels and visualized using a BioRad Gel-Doc XR + gel-documenting system (Bio-Rad, Hercules, CA, USA).

## Results

**Fig. 1:**
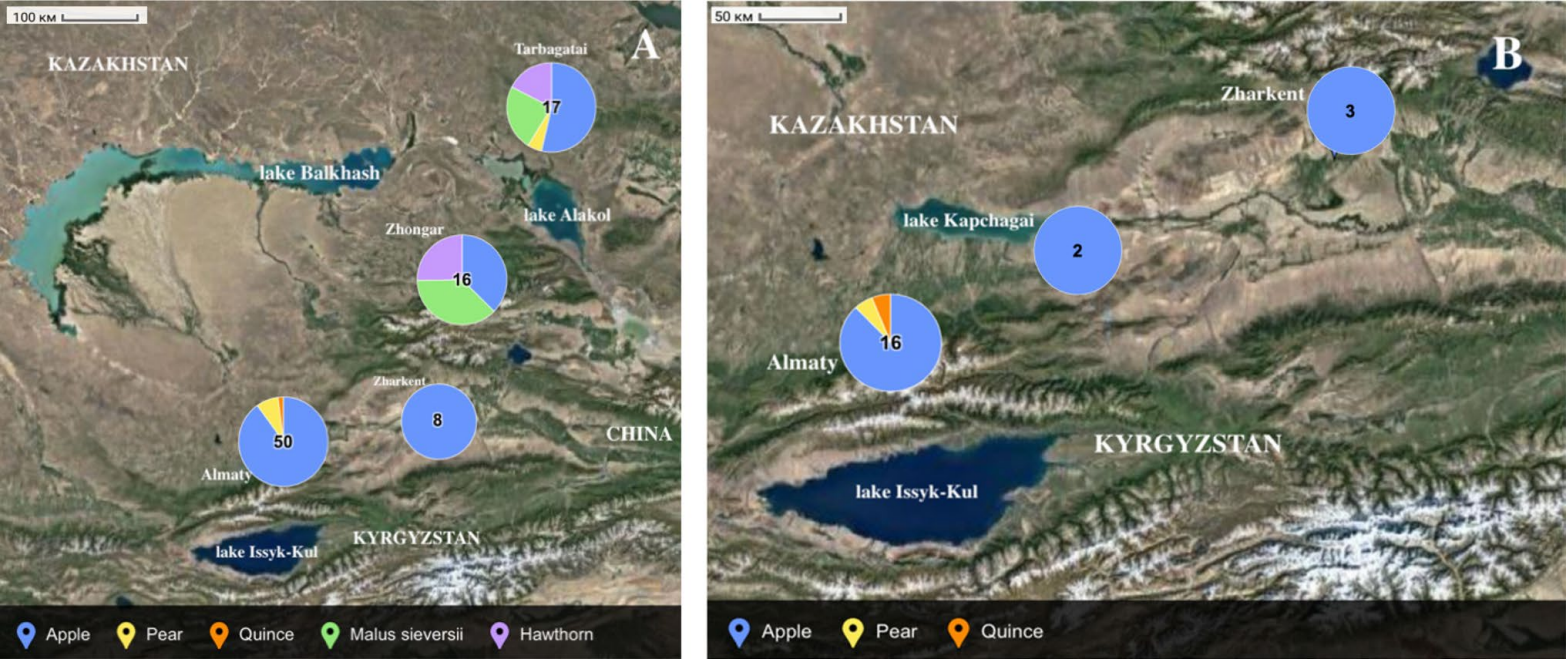
Sampling and effective isolates. **A** Host species composition of the samples collected in 2022, clustered in four collection zones. **B** Location of the isolates obtained in southeastern Kazakhstan in 2022

The phytopathological observations were conducted in three regions (oblasts) and one city of Kazakhstan in small and medium size orchards (not larger than 25 ha), private households, public locations, and in stands and forests of *M. sieversii* in Ile-Alatau and Tarbagatai state national nature parks (Table 2). Samples with symptoms not excluding fire blight (brown and black shoots, flowers and fruitlets, bended shoot tips—“shepherds crook”), or asymptomatic in case of wild apple and hawthorn trees, were collected from each site. A total of 91 samples were collected, including 68 samples from cultural apple, ten from wild apple (*M. sieversii*), five from pear, seven from hawthorn, and one from quince (Table 3). The collected samples could be clustered into four major sampling areas, being the greater Almaty area, Zharkent area, Zhongar area, and Tarbagatai area (Fig. 1A).

**Table 2:** Sampling locations and disease indices, number of collected plant samples, and obtained isolates

Sampling area	Number of samples collected	Number of isolates obtained	Area inspected (ha)	Average disease indices	
				*P*^a^	*R*^a^
Greater Almaty area	50	18	52,92	4,5	0,9
Zharkent	8	3	97,7	6,3	1,5
Zhongar	16	0	7,5	2,5	0,5
Tarbagatai	17	0	17	0,9	0,1
Total	91	21	175,1		

^a^*P* percent of distribution (frequency of occurrence), *R* degree of disease development

**Table 3:** Host species composition of the plant samples collected in the field in 2022

Sampling area	*M. domestica*	*M. sieversii*	Pear	Other	Total
Greater Almaty area	45	0	4	1 (quince)	50
Zharkent area	8	0	0	0	8
Zhongar	6	6	0	4 (hawthorn)	16
Tarbagatai	9	4	1	3 (hawthorn)	17

**Fig. 2:**
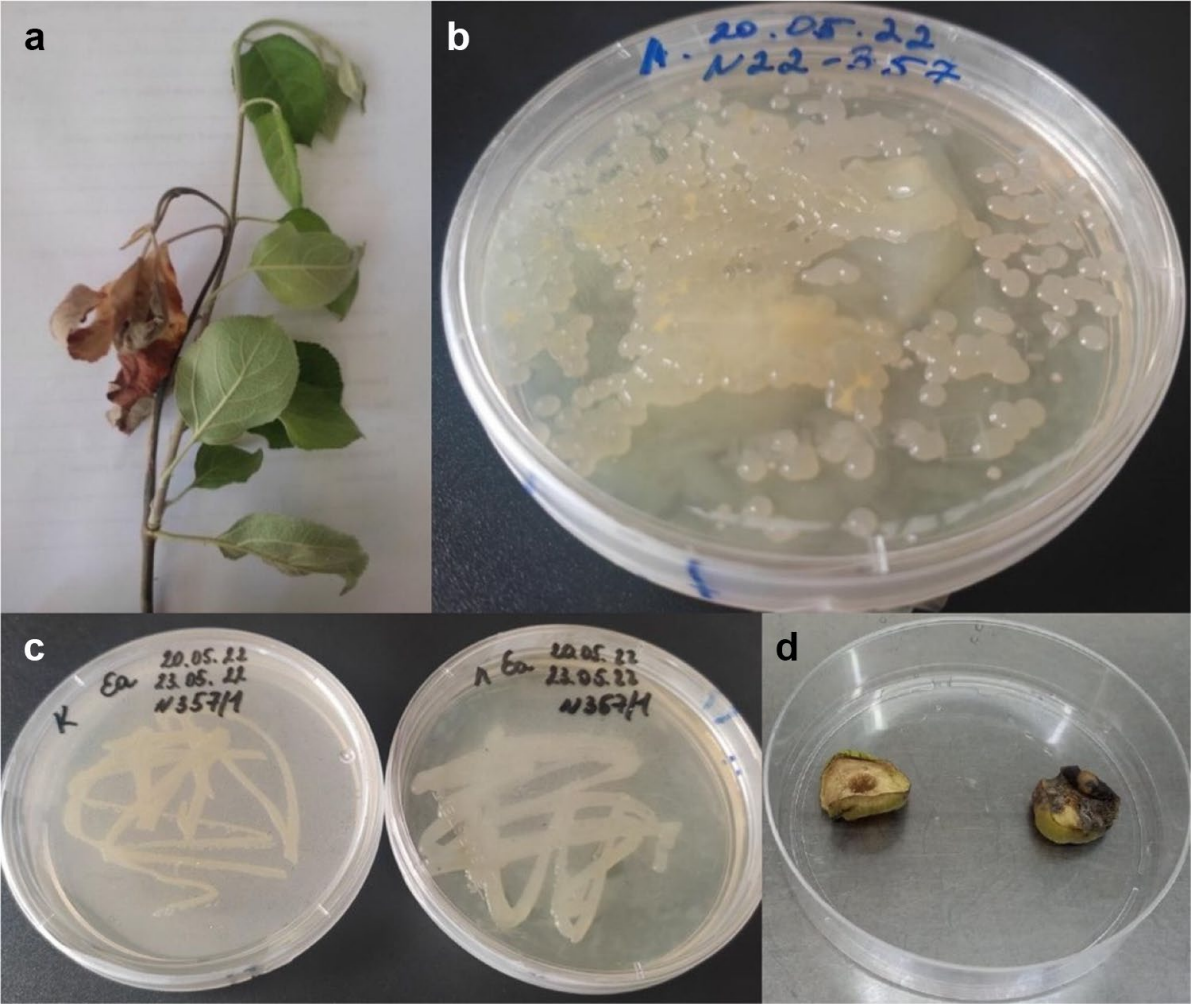
Laboratory processing cycle of a collected plant sample. **a** Plant sample with symptoms (in the photo – browning and bending of the cultural apple shoot’s tip); **b** mixed culture obtained on levan medium; **c** pure culture on King’s B (left) and levan (right) media; **d** immature pear fruit assay

Symptoms such as shoot darkening and bending (“shepherds crook”), wilting, and fruitlets mummification were recorded. These symptoms are compatible with fire blight, so all the samples showing at least one of these symptoms were collected for further laboratory confirmation. The 21 samples yielding colonies with distinct *E. amylovora* characteristics (milky white or yellowish domed, circular, smooth colonies on levan medium while forming white mucoid colonies on King’s B agar with no fluorescence under UV light) were subcultured to obtain pure cultures (Fig. 2).

Among the isolates obtained in this study, one originated from pear, one from quince, and the remaining 19 isolates were sampled from cultivated apple (Table 4). Both quince and pear were collected in Almaty city. Three isolates from apple host were obtained from Zharkent sampling area and the other sixteen from the greater Almaty area (Fig. 1B). No isolates nor positive molecular and bacteriological results were obtained for samples from Zhongar and Tarbagatai sampling areas.

**Table 4:** The list of *Erwinia amylovora* isolates obtained in 2022 in southeastern Kazakhstan (Almaty oblast) with genotyping information

Strain name	Origin	District, village	Host species	Agristrip^a^	PCR^a^	Immature pear fruitlet assay	CRISPR genotype^b^
KZ346	Orchard	Panfilov, Pidzhim	*M. domestica* var. Golden Delicious	+	+	+	A
KZ348	Orchard	Enbekshikazakh, Chilik	*M. domestica* var. Golden Delicious	+	+	+	A
KZ350	Orchard	Enbekshikazakh, Chilik	*M. domestica* var. Zarya Alatau	+	+	+	A
KZ358	Orchard	Panfilov, Pidzhim	*M. domestica* var. Aport	+	+	+	A
KZ359	Orchard	Panfilov, Pidzhim	*M. domestica* var. Aport	+	+	+	A
KZ362	Orchard	Almaty city	*M. domestica* var. Aport	+	+	+	A
KZ363	Orchard	Almaty city	*M. domestica* var. Voskhod	+	+	+	A
KZ364	Orchard	Almaty city	*M. domestica* var. Aport	+	+	+	A
KZ366	Orchard	Almaty city, Talgar	*M. domestica* var. Aport	+	+	+	A
KZ367	Orchard	Almaty city, Talgar	*M. domestica* var. Aport	+	+	+	A
KZ368	Orchard	Almaty city, Talgar	*M. domestica* var. Aport	+	+	+	A
KZ369	Orchard	Almaty city, Talgar	*M. domestica* var. Aport	+	+	+	A
KZ370	Orchard	Almaty city, Talgar	*M. domestica* var. Aport	+	+	+	A
KZ374	Orchard	Almaty city, Talgar	*M. domestica*	+	+	+	A
KZ376	Orchard	Almaty city, Talgar	*M. domestica* var. Aport	+	+	+	A
KZ378	Orchard	Almaty city	*M. domestica*	+	+	+	A
KZ379	Orchard	Almaty city	*M. domestica*	+	+	+	A
KZ380	Orchard	Almaty city	*M. domestica*	+	+	+	A
KZ382	Orchard	Almaty city	*M. domestica*	+	+	+	A
KZ384	Orchard	Almaty city	*P. communis* var. Talgar Beauty	+	+	+	A
KZ386	Orchard	Almaty city	*Cydonia* sp.	+	+	+	A

^a^+, positive for *E. amylovora* by both Stöger’s and Pirc’s protocols

^b^As according to Kurz et al. (2021)

After the species was positively identified as *E. amylovora* by classic PCR and immunochromatographic methods (Bioreba Agristrip test; Table 4), the pure cultures were checked for pathogenicity using the immature pear fruitlet assay. All pure cultures with positive results shown by molecular and bacteriologic methods provided positive immature pear fruitlet results, forming milky ooze, which was re-tested with molecular methods and further prepared for long-term storage.

**Fig. 3:**
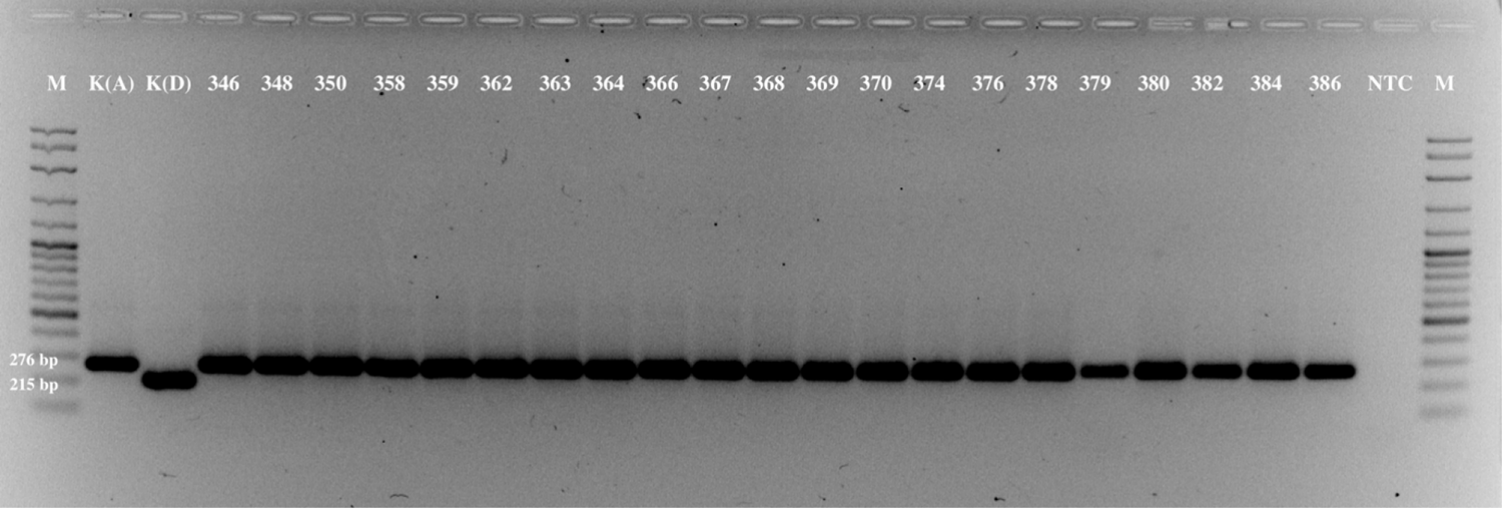
Molecular analysis of the isolates obtained in southeastern Kazakhstan in 2022 for duplication of CRISPR spacer 1029 (weight increments of the molecular marker (M) is 100 bp. K(A), *E. amylovora* CFBP 1430; K(D), *E. amylovora* CFBP 3098; NTC, non-template control

The phylogenetic studies of *E. amylovora* have shown high genetic homogeneity within isolates of the widely prevalent group (Kurz et al. 2021; Parcey et al. 2020; Zeng et al. 2018). One of the approaches in the characterization of strains is the analysis of hypervariable regions in clustered regularly interspaced short palindromic repeats (CRISPR) (Rezzonico et al. 2011). The analysis of the isolates collected in southeastern Kazakhstan in 2022 revealed that all samples belong to archetypal genotype A like strain CFBP 1430 (Fig. 3), indicating that all strains have the duplication of CRISPR spacer 1029 in CRR1 as observed before in Kazakhstan and in the surrounding Central Asian countries (Doolotkeldieva et al. 2021; Kurz et al. 2021).

## Discussion

Fire blight of apples and other pome fruits is present in Kazakhstan since about a decade. The initial unpreparedness and lack of awareness were partially compensated by a set of legal acts that strengthened import control and provided fruit growers with state-funded fire blight treatment preparations and reimbursement opportunities in case a tree had to be eradicated to prevent further spread of the disease. Nevertheless, an awareness-raising campaign is still needed for owners of small orchard and private households, which are sometimes overlooked in more agricultural production-oriented state programs.

Unlike the past several years, the first half of 2022 was favorable for fire blight development due to weather conditions, and the disease was detected in various locations not too far from the growth zone of wild apples. The distance of 20–30 km that separates the infected trees from their wild relatives still leaves some room for action. Although no wild apple and hawthorn trees observed in this study showed any symptoms, urgent measures need to be developed to not let this distance decrease with time. In accordance with the state law, there are 2-km buffer zones surrounding the protected areas, but these may be largely insufficient in case of a pathogen such as *E. amylovora* that is able to cross these relatively short distances with the help of insects and birds. One underestimated threat is constituted by the local population that can dramatically shorten the distance between infected and healthy zones by carrying the pathogen on themselves (via infected fruits or twigs, clothes, utensils, etc.). This is also the reason a special awareness-raising campaign and training is needed *inter alia* for the rangers in the specially protected natural territories aimed at the safeguard of wild apple trees. The preparedness of these specialists is key to monitoring and timely prevention of fire blight spread.

The location of the *E. amylovora*-positive samples around Almaty was quite predictable. Almaty is the largest city of Kazakhstan situated in the apple-growing zone with many people purchasing imported apple trees for personal use in their households and summer houses. These results also correspond to the analysis of large fire blight foci distribution in commercial orchards in 2018 (Umiraliyeva et al. 2021), where the majority of the foci was registered in the commercial orchards in Almaty city area. On the other hand, the Zhongar and Tarbagatai areas are not as densely populated and the local population has less opportunities to purchase imported plant material for private households and small orchards, thus halting the spread of the disease to these areas.

Only 21 out of 91 samples proved that the observed symptoms were due to infection with *E. amylovora*. This is explained by the strategy of the study, where all the samples are taken with the symptoms that could not exclude fireblight, even if they could be attributed to other diseases and factors, in order to not miss the disease.

Archetypal genotype A, found in all the isolates, can help in speculatively tracking the origin of those isolates to northwestern Europe or the Mediterranean area, where this genotype prevails (Kurz et al. 2021) and from where large batches of planting material were imported. To date, mostly A-derived genotypes were registered in Kazakhstan (Doolotkeldieva et al. 2021; Kurz et al. 2021), congruent to the results of the current study. Nevertheless, evidence is available that archetypal genotype D was present in South Kazakhstan as early as 2014 **(**Rezzonico, Drenova and Smits, unpublished results).

Although the country promptly reacted to the fire blight introduction, further introduction events and spread of the disease within the orchards of small farmers and household owners have been and will be inevitable due to massive import of improperly tested planting material. It is crucial to protect the wild apple forests against the potential threat that fire blight is posing, and special prevention efforts should be taken timely.

## Data Availability

The authors declare that the data supporting the findings of this study are available within the article and its supplementary material; additional data is available from the corresponding author on reasonable request.
